# Water quality assessment of east Tiaoxi River, China, based on a comprehensive water quality index model and Monte-Carlo simulation

**DOI:** 10.1038/s41598-022-14293-9

**Published:** 2022-06-16

**Authors:** Wei Jin, Yuan Li, Li Lu, Dong Zhang, Shanying He, Jiali Shentu, Qiwei Chai, Lei Huang

**Affiliations:** 1 Zhejiang Provincial Key Laboratory of Solid Waste Treatment and Recycling, School of Environmental Science and Engineering, Zhejiang Gongshang University, Hangzhou, 310018 China; 2 Instrumental Analysis Center of Zhejiang, Gongshang University, Hangzhou, 310018 China; 3 College of Materials and Environmental Engineering, Hangzhou Dianzi University, Hangzhou, 310018 China

**Keywords:** Environmental chemistry, Environmental impact

## Abstract

The comprehensive water quality index (*CWQI*) reflects the comprehensive pollution status of rivers through mathematical statistics of several water quality indicators. Using computational mathematical simulations, high-confidence *CWQI* predictions can be obtained based on limited water quality monitoring samples. At present, most of the *CWQI* reported in the literature are based on conventional indicators such as nitrogen and phosphorus levels, and do not include the petroleum hydrocarbons levels. This article takes a typical river in eastern China as an example, based on the 1-year monitoring at 20 sampling sets, a *CWQI* containing five factors, TN, NH_4_^+^-N, TP, ∑n-Alks, and ∑PAHs was established, and further predicted by a Monte-Carlo model. The predicted *CWQI* for each monitoring section is above 0.7, indicating that most of the monitoring sections are moderately polluted, and some sections are seriously polluted. The Spearman rank correlation coefficient analysis results show that TN, ∑PAHs, and ∑n-Alks are the main factors influencing the water quality, especially the petroleum hydrocarbons have a significant impact on the middle and lower reaches due to shipping. In the future, more attention should be paid to petroleum hydrocarbon organic pollutants in the water quality evaluation of similar rivers.

## Introduction

River as important fresh water resources on land, bear important missions in people's daily life, but at the same time, river pollution, as a serious problem worldwide, threatens environmental safety and people's healthy^1^. The collection of river water quality related data can be used as the basis for river management^2^. Collection of data on river water pollutants is generally based on several aspects. The first is an indicator system based on eutrophication of water bodies, especially nitrogen, phosphorus, dissolved oxygen and other indicators have important impacts on river ecology. River assessment system based on indicators is widely used^3–5^. The second is about trace pollutants including PAHs, antibiotics, etc. These pollutants are extremely low in water, but their hard-to-degrade and cumulative properties will undoubtedly affect the ecological environment at a higher concentration^6,7^. It has an important impact, and the collection of trace pollutant data has a longer-term direction for the formulation of river ecological management plans. The other is the collection of some related indicators of particulate matter and microorganisms. After a thorough understanding of the concentration level of pollutants in the basin, water quality assessment can be used to simulate the assessment of the pollution situation in the area. As a kind of water quality index (*WQI*), The Comprehensive Water Quality Index (*CWQI*) Method has the advantages of being able to process long series of data and comprehensive water quality for multi-point and multi section water bodies^8^. *CWQI* assigns water quality levels based on pollution classification standards. *CWQI* is in the range of (0, 0.4], which is a clean water body, and the water quality is better; in the range of (0.4, 0.7], the water quality is slightly pollution; in (0.7, 1], the water quality is moderate pollution; (1, 2], the water quality is a serious pollution; at (2, + ∞), the water quality pollution is very serious^9^. The selected water quality index will be calculated according to the following formula:

1 $$CWQI=\frac{1}{n}\sum_{i=1}^{n}{P}_{i}$$

In the formula, n is the number of participating water quality indicators; P_i_ is the single factor pollution index. The larger the value, the higher the degree of pollution.

For non-dissolved oxygen indicators:

2 $${P}_{i}={C}_{i}/{C}_{0}$$

In the formula, C_i_ is the measured value of an evaluation index; C0 is the standard value of an evaluation index.

Farzadkia et al.^10^ used *CWQI* to model 14 pollutant indicators, including (temperature, electrical conductivity (EC), turbidity, total dissolved solids (TDS), total suspended solids (TSS), nitrate, nitrite, phosphate, dissolved oxygen (DO), chemical oxygen demand (COD), biochemical oxygen demand (BOD), pH, and total coliforms (TCs) and fecal coliforms (FCs). Use this to assess the water quality of the Yamchi Dam basin and identify the main source of water pollution in the basin. Davies et al.^11^ used *CWQI* simulation analysis was performed under the conditions of 17 pollutant indicators (including TP, NH_4_^+^-N, DO, etc.), and it was found that the value of *CWQI* can reflect the change of the sample. It can be seen that *CWQI* is mainly targeted at conventional indicators in previous studies, and *CWQI* can only obtain corresponding water quality conditions from monitoring data during use. Once the number of samples is too small, the significance of its evaluation will be greatly reduced.

The Monte Carlo model can make up for the shortcomings of *CWQI* when the amount of data is insufficient. Monte Carlo simulation belongs to a branch of computational mathematics. Through random sampling, the average value of a random variable or the probability of this event occurring is used as a solution to the problem. By performing thousands of Monte Carlo sampling simulations on the basic data of river water quality obtained by sampling a limited number of times, all possible comprehensive pollution indexes and their probabilities can be obtained under uncertain conditions of river water quality evaluation parameters, and the credibility and statistics of the simulation results Significantly higher than the result obtained from a limited number of samples^12^.

In this study, 20 sampling sites were set up in East Tiaoxi River to collect data for one year. And a Monte Carlo-*CWQI* model with 5 pollutant indicators including TN, NH_4_^+^-N, TP, ∑n-Alks, ∑PAHs is established to evaluate the water quality. The aim of this article is to further improve the database of pollutants in Esat Tiaoxi River and analyze the comprehensive pollution level of the river and obtain the main pollutants and main influencing factors.

## Material and methods

### Study area

**Figure 1:**
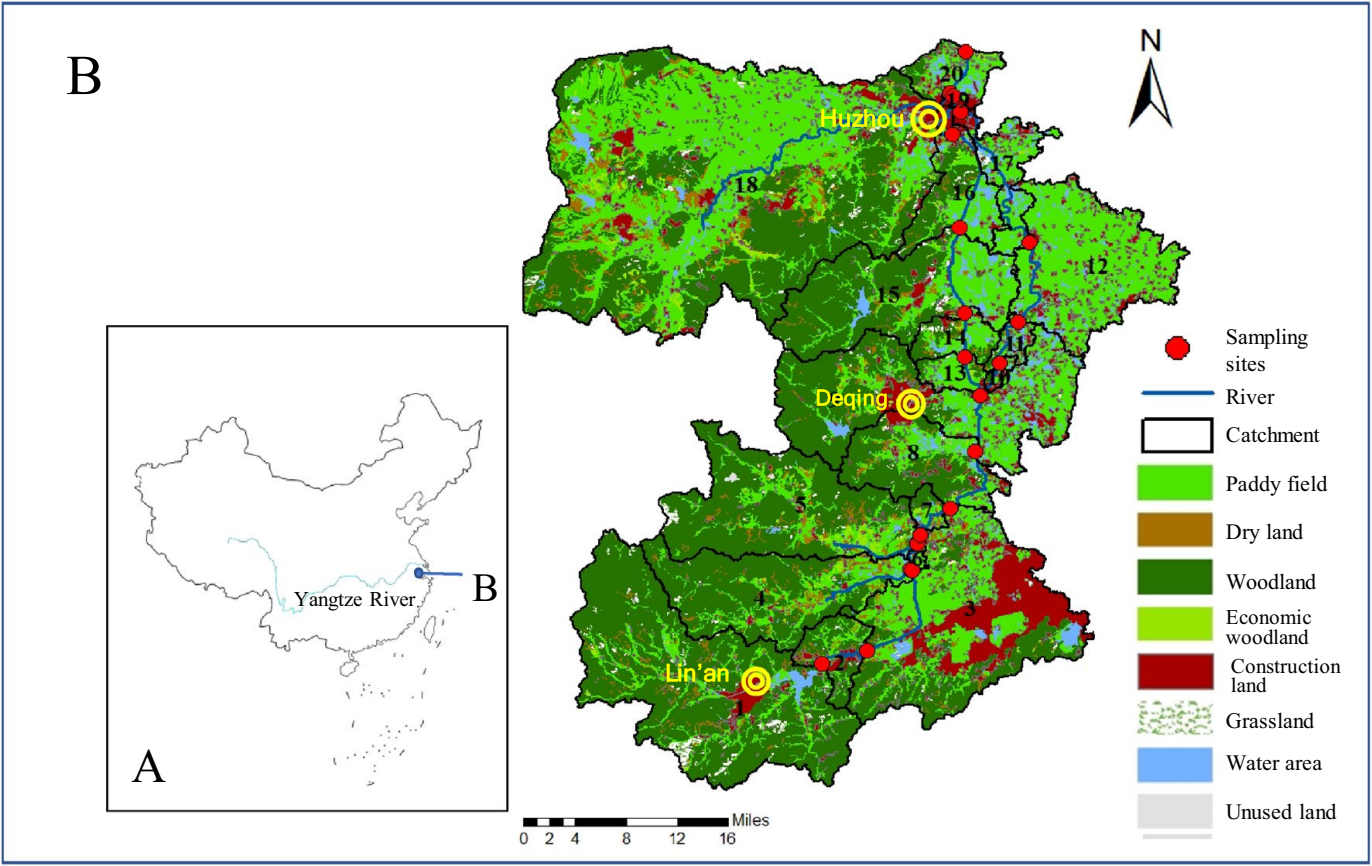
The catchments and sampling points (ArcGIS10.1, ESRI).

The East Tiaoxi River is a typical basin of the river network in Southeast China. It is one of the largest inflow systems of Lake Taihu. The length of the main stream is 151.4 km (30° 05′ ~ 30° 57′ N, 119° 28′ ~ 120° 08′ E), and the basin area reaches 2267 km^2^ (Fig. 1). The upper stream of East Tiaoxi River is a mountain river, which is composed of three tributaries of North Tiaoxi River, Mid Tiaoxi River, and North Tiaoxi River. The middle reaches of the river are narrow and are flooded areas. The lower reaches of the Hangjiahu Plain, with wide rivers, slow rivers, and advanced shipping. It is an important shipping area with a developed fish breeding industry. As an important water source in this region, the water quality of East Tiaoxi River is related to the ecological security of the basin and people's health.

According to the distribution of water systems in the basin, we use GIS (ArcGIS 10.1, ESRI Company, Redlands, California, USA) to divide the whole basin into 20 catchments, and set up a water quality monitoring section on the main channel in each catchment (Fig. 1). A total of 20 sampling points (set to P1–P20) were set, including 6 tributary monitoring points (P1; P2; P3; P4; P5; P18) and 14 main stream monitoring points. The land type data and population data of each catchment were collected and listed in Supplementary Materials (Table S1).

### Sample collection and laboratory analysis

From September 2018 to August 2019, monthly sampling of 20 river water quality monitoring sections of East Tiaoxi River was conducted. Each month, sample collection for all sections was completed in two consecutive daytime hours. The surface water sample is collected in the middle of each monitoring section and 0.5 m away from the water surface, each sample is 10 L. After the samples were collected, they were acidified by adding sulfuric acid to a pH of less than 2 and shaken and stored in a refrigerator.

Standard Methods recommended by Ministry of Ecology and Environment of China were used for ammonia nitrogen (NH4-N, HJ 535-2009), total nitrogen (TN, HJ 636-2012), and total phosphorus (TP, GB 11893-89) analysis. Specifically, NH4-N was determined using a Nessler’s reagent spectrophotometric method; TN was determined using an alkaline potassium persulfate digestion UV spectrophotometric method; TP was determined using ammonium molybdate spectrophotometric method^13,14^. Water samples of 16 kinds of PAHs were passed through a LC-C18 cartridge for solid-phase extraction (SUPELCO Visiprep), eluted with dichloromethane, and after nitrogen blowing, the volume was adjusted with acetonitrile and determined by High Performance Liquid Chromatography (Agilent 1260, USA)^15^. 33 kinds of n-Alkanes (C8–C40) water samples were also passed through a LC-C18 cartridge for solid phase extraction and eluted with ethyl acetate. After nitrogen blowing, they were further eluted through a silica gel column with n-hexane and then nitrogen Blow, make up to volume with n-hexane, and measure by Gas Chromatography (Agilent 7890, USA)^16^.

### Monte Carlo-CWQI Index model and spearman correlation coefficient

In this study, Monte Carlo simulation was used, combined with *CWQI*, *CWQI* was used as a predictive variable, and random variables were set as P_i_ for each evaluation index to establish a Monte Carlo-*CWQI* water quality evaluation model. The methods of establishing the comprehensive pollution index and Monte Carlo simulation are introduced in “The comprehensive pollution index method” and “Monte Carlo simulation method”, respectively.

#### The comprehensive pollution index method

Based on the monitoring data of the above five pollutants, we established a comprehensive pollution index model for TN, NH_4_^+^-N, TP, ∑n-Alks and ∑PAHs, and established the standard values of the single factor indicators for the five pollutants according to the formula (2). TN, NH_4_^+^-N and TP are based on the standard value of class III water in environmental quality standards for surface water in China (GB3838-2002). Compared with some European standards, the concentration of TN we set is at the same level as that of the category III (0.75 ~ 1.5 mg L^−1^) of the UNECE (the United Nations Economic Commission for Europe) ambient water quality standard, and the concentration of NH_4_^+^-N is between the level of class III and class IV of the ICPDR (International Commission for the Protection of the Danube River) water quality classification (0.6 ~ 1.5 mg L^−1^). The concentration of TP is at the ICPDR standard class II level (0.2 mg L^−1^)^17^. In the U.S., EPA divided the whole area into 14 distinct eco-regions, and proposed recommended ambient water criteria for each region. The TN standard selected here is comparable to the higher levels in the 14 regions (eg. eco-region XII, 0.9 mg L^−1^), while the TP standard is significantly lower than those in all 14 regions^18^.

The standard value of ∑n-Alks here is based on the standard value of class III water in petroleum hydrocarbons in GB3838-2002. However, prior to this study, there is no suitable standard value for ∑PAHs. In this study, the standard value of benzoapyrene (BaP) 2.8 ng L^−1^ in GB3838-2002 was expanded by a hundred times (280 ng L^−1^) as the standard value for ∑PAHs. The specific standard values are shown in Table 1.

**Table 1:** Calculated standard values of the five single-factor pollution indexes.

Index	TN	NH_4_^+^-N	TP	∑PAHs	∑n-Alks
Unit	mg L^−1^	mg L^−1^	mg L^−1^	ng L^−1^	μg L^−1^
Standard value	1.0^a^	1.0^a^	0.2^a^	280^b^	50^c^

^a^The standard value of class III water in environmental quality standards for surface water in China (GB3838-2002).

^b^One hundred times the standard value of 2.8 ng L^−1^ of benzo(a)pyrene (BaP) in GB3838-2002.

^c^The standard value of class III water in petroleum hydrocarbons in GB3838-2002.

#### Monte Carlo simulation method

Based on the standard values of the above indicators, the single factor pollution index of each indicator is calculated by the formula (2). In order to ensure that the data is in a normal distribution, the obtained single-factor pollution index is subjected to natural logarithmic transformation, and SPSS 22.0 is used for Kolmogorov–Smirnov test. The relevant data of the single factor pollution index can be found in the supplementary materials (Table S2). Monte Carlo simulation was performed in Crystal Ball software in the Microsoft Excel environment. Edit the formula (1) in the Monte Carlo model, use *CWQI* as the predictive variable, and use the single factor pollution index as the random variable. Enter the mean and standard values of the single-factor pollution index into the Monte Carlo model and perform 10,000 sampling simulation.

In order to characterize the impact of various water quality indicators on the degree of water pollution, Spearman rank correlation coefficient (*SRCC*) will be calculated according to the following formula:

3 $$SRCC=\frac{\sum_{i=1}^{m}({x}_{i}-\overline{x })({y}_{i}-\overline{y })}{{[\sum_{i=1}^{m}{({x}_{i}-\overline{x })}^{2}\sum_{i=1}^{m}{({y}_{i}-\overline{y })}^{2}]}^{1/2}}$$

In the formula, m is the number of simulations; x_i_ is the ranking value of the input parameter; y_i_ is the ranking value of the output result; $\overline{x }$ and $\overline{y }$ are the means of the x_i_s and the y_i_s. The value of *SRCC* ranges from − 1 to 1. The higher the *SRCC* value, the greater the influence of the input variable on the target variable.

## Results and discussion

### Concentration levels and distribution characteristics of the typical pollutants

**Figure 2:**
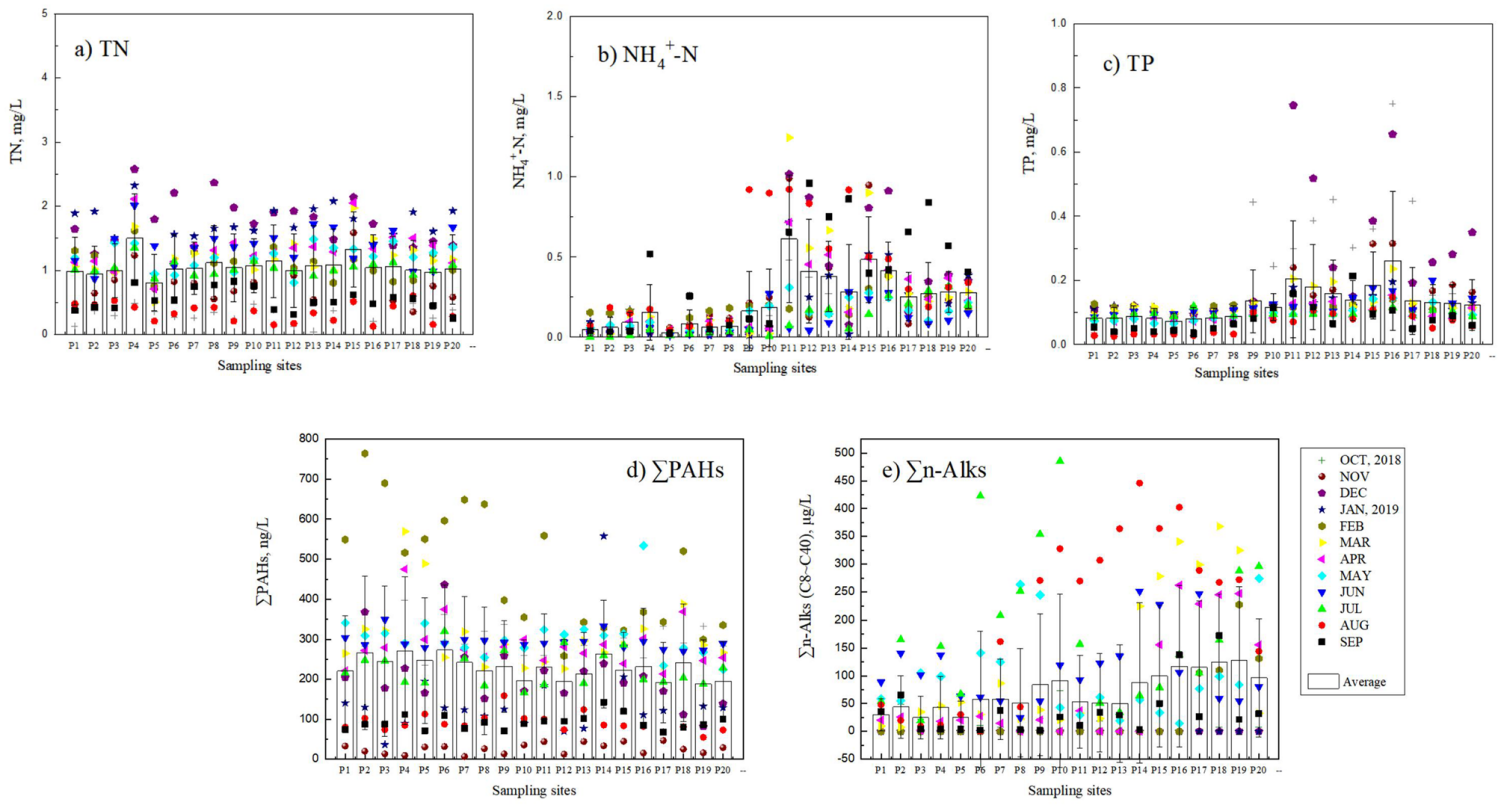
Annual average concentration and distribution of the typical pollutants in each monitoring section along the East Tiaoxi River. (**a**) TN, (**b**) NH_4_-N, (**c**) TP, (**d**) ∑PAHs, (**e**) ∑n-Alks (C8 ~ C40).

According to the monitoring results, the annual average concentration of TN, NH_4_^+^-N are 0.80–1.50 mg L^−1^ and 0.03–0.61 mg L^−1^, respectively. The TN concentration in East Tiaoxi River is relatively stable throughout the year, with a high concentration in December and an average concentration of 1.74 mg L^−1^ in the basin (Fig. 2a). The variation trend of TN concentration in the river within one year is basically consistent with the change of runoff. The highest annual concentration appears in P4 of Mid Tiaoxi River monitoring section, with an average concentration of 1.50 mg L^−1^ and a maximum concentration of 2.58 mg L^−1^. There may be a large number of pollution sources of fertilization or discharge in this area. The highest annual concentration of NH_4_^+^-N is found in monitoring section P11, with an average concentration of 0.61 mg L^−1^ and a maximum concentration of 1.24 mg L^−1^. Since monitoring section P11, the concentration of NH_4_^+^-N in East Tiaoxi River began to rise, and the concentration in the middle and lower reaches of East Tiaoxi River was generally higher than that in the middle and upper reaches; the concentration of NH_4_^+^-N reached the highest in August and September, with an average concentration of 0.40 mg L^−1^ and 0.38 mg L^−1^ (Fig. 2b).

The annual average concentrations of TP are 0.07 ~ 0.26 mg L^−1^. The highest annual concentration of TP is found in monitoring section P17 at the lower reaches of East Tiaoxi River, with an average concentration of 0.13 mg L^−1^ and a maximum concentration of 0.44 mg L^−1^. The abnormal value of TP occurred in December 2018. From monitoring section P11 to monitoring section P20 at the inlet of the lake, the concentration increased to varying degrees, with an average concentration of 0.24 mg L^−1^(Fig. 2c).

In the monitoring of 16 kinds of PAHs, it is found that the ∑PAHs s in each monitoring section of East Tiaoxi River basin is 0.19–0.28 μg L^−1^, which is lower than other rivers in China^19–21^, but the analysis on time scale shows that the concentration of PAHs in February is generally high, with an average concentration of 0.47 μg L^−1^, and some sections also have high concentrations in January and March. To determine the source of PAHs, more long-term monitoring is needed (Fig. 2d).

As the middle and lower reaches of East Tiaoxi River are widely used in shipping, n-alkanes (the n-alkanes with carbon chain lengths from 8 to 40, which are denoted by C8 to C40) was monitored to explore the impact of shipping on water quality. In the monitoring of n-alkanes (C8 ~ C40), it was found that ∑n-Alks concentration of 20 monitoring sections in East Tiaoxi River was 24.92–127.10 μg L^−1^. In terms of average concentration, the areas with higher concentration are mainly concentrated in the middle and lower reaches of East Tiaoxi River, which coincides with the shipping area. In terms of time, the high concentration points are mainly in July and August, with the average concentrations of 178.02 μg L^−1^ and 202.42 μg L^−1^. At this time, the rainfall is large, the water level rises, and the shipping is relatively frequent (Fig. 2e).

### Analysis of types and characteristics of the organic pollutants

**Figure 3:**
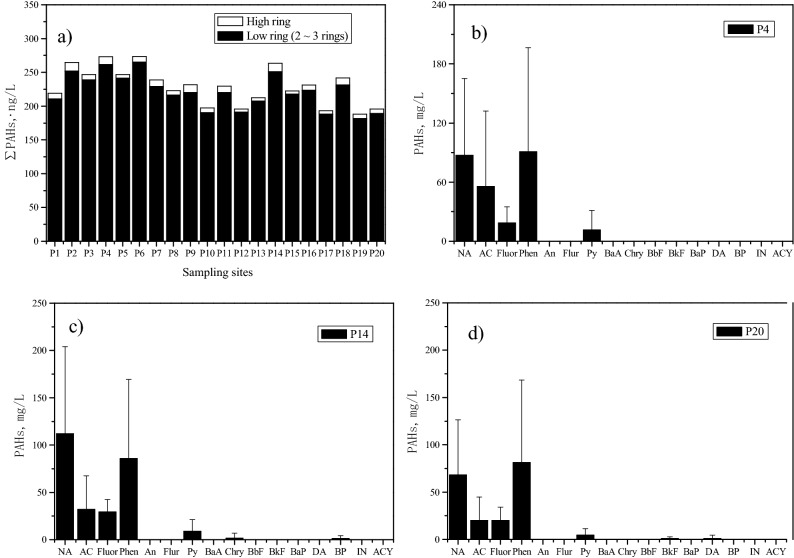
Distribution characteristics of the 16 PAH in the monitoring sections of P4, P14 and P20. (**a**) ∑PAHs, (**b**) P4, (**c**) P14, (**d**) P20.

The characteristics of 16 types of PAHs were analyzed. Overall, the low loops (2 to 3 loops) accounted for about 98% of the total PAHs (Fig. 3a). Three monitoring sections (P4, P14 and P20) were selected in the upstream, middle and lower reaches of East Tiaoxi River for more detailed analysis. NA and Phen are the PAHs that mainly exist in East Tiaoxi River. AC, Flour, and Py also exist. The concentration of Phen in the river basin as a whole did not change much. The concentration of NA in the upper and middle reaches is higher, which may be caused by the disturbance of the river in the upper and middle reaches. The concentration of AC is higher in the upper reaches of East Tiaoxi River (Fig. 3b–d).

Three monitoring sections (P2, P15 and P20) were also selected for the analysis of 33 types of n-alkanes in the upper, middle and lower reaches of East Tiaoxi River. C22 is the main pollutant in the upstream region of East Tiaoxi River, and C39 is the main pollutant in the middle and lower reaches (Supplementary Fig. S1). From the perspective of monthly distribution, the production of high-density n-alkanes in some months is the cause of excessively high n-alkanes concentration in the East Tiaoxi River.

The carbon preference index (*CPI*) was selected to conduct a qualitative analysis of the origin of n-alkanes in the East Tiaoxi River. *CPI* is the concentration ratio of the sum of odd-numbered alkanes to the sum of even-numbered alkanes. When *CPI* > 5, n-alkanes comes from plant wax source; when *CPI*≈1, n-alkanes is considered to come from fossil fuel^22,23^.

4 $$ {\text{CPI}} = \left( {{\text{C}}9 + {\text{C}}11 + {\text{C}}13 + {\text{C}}15 + {\text{C}}17 + {\text{C}}19 + {\text{C}}21 + {\text{C}}23 + {\text{C}}25 + {\text{C}}27 + {\text{C}}29 + {\text{C}}31 + {\text{C}}33 + {\text{C}}35 + {\text{C}}37 + {\text{C}}39} \right)/\left( {{\text{C}}8 + {\text{C}}10 + {\text{C}}12 + {\text{C}}14 + {\text{C}}16 + {\text{C}}18 + {\text{C}}20 + {\text{C}}22 + {\text{C}}24 + {\text{C}}26 + {\text{C}}28 + {\text{C}}30 + {\text{C}}32 + {\text{C}}34 + {\text{C}}36 + {\text{C}}38 + {\text{C}}40} \right) $$

According to the formula (4), the *CPI* values of the monitoring sections P2, P15, and P20 are 1.37, 2.43, and 1.94, respectively. The *CPI* is higher than 1 but much lower than 5, and the n-alkanes in the East Tiaoxi River mainly come from fossil fuels.

### Monte Carlo-CWQI for water quality evaluation

According to the water quality monitoring data of each section, a series of single-factor pollution index values were calculated by formula (2) and then the natural logarithmic transformation was performed. After the Kolmogorov–Smirnov test, the transformed single-factor pollution indices all obeyed normal distribution, and their mean values and probability distribution parameters are shown in Table S2.

Although the number of sampling times for water quality monitoring of any river is always limited, Monte Carlo simulation can obtain all possible comprehensive pollution indices and parameters under uncertain conditions of river water quality evaluation parameters by randomly sampling the limited basic data^24–26^. In this study, the above-mentioned single-factor pollution index was set as a random variable, and the probability distribution parameters of each random variable were input. For each section, randomly sample 10,000 times within the range of the probability distribution, and the cumulative frequency distributions of *CWQI* for 20 monitoring sections, the *CWQI* prediction average value, and the probability of different degrees of pollution in each monitoring section can be obtained (Table. 2).

**Table 2:** Probability distributions of the *CWQI* for the different sampling locations in the East Tiaoxi River.

Sampling sites	Average predicted *CWQI* value	Clean water (%)	Slight pollution (%)	Moderate pollution (%)	Serious pollution (%)	Highly severe pollution (%)
P1	0.76	1	47	38	13	1
P2	0.89	0	28	44	26	2
P3	0.84	1	40	38	19	2
P4	0.98	0	25	39	33	3
P5	0.72	1	54	35	9	1
P6	0.81	0	40	42	17	1
P7	0.82	0	45	37	16	2
P8	0.78	0	43	44	12	1
P9	0.88	0	30	44	24	2
P10	0.86	0	26	52	22	1
P11	1.08	0	10	39	48	3
P12	1.00	0	12	46	40	2
P13	0.98	0	22	44	31	3
P14	1.03	0	17	38	42	3
P15	1.14	0	6	34	57	3
P16	1.13	0	10	34	29	4
P17	0.95	0	20	45	34	1
P18	0.94	0	18	49	32	1
P19	0.90	0	25	47	27	1
P20	0.84	0	29	52	18	1

**Figure 4:**
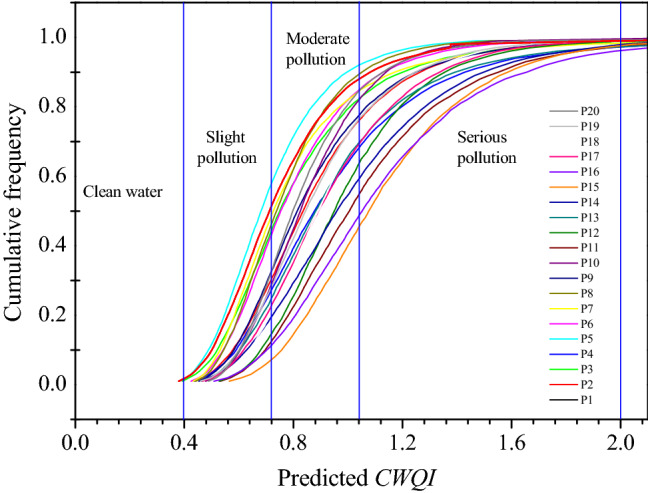
Cumulative probability curve of the *CWQI* for the different sampling locations in the East Tiaoxi River.

The results show that the average predicted *CWQI* of each monitoring section in East Tiaoxi River is above 0.7 (Table. 2). The water quality in North Tiaoxi River (monitoring section P5) is the best. Monte Carlo simulation shows that the probability of water pollution in monitoring section P5 is 54%, and the average *CWQI* prediction is 0.72. The water quality in the middle and lower reaches of the East Tiaoxi River is significantly inferior to that in the upper reaches. Judging from the predicted average values, the predicted *CWQI* average values of monitoring sections P11–P12 and P14–P16 are all greater than 1, which is a serious pollution. Among them, the probability of severe pollution in monitoring section P15 reached 57%, and the predicted average value of *CWQI* was 1.14. The water quality in this area was the worst. In summary, the overall water quality of the East Tiaoxi River is relatively poor, mainly with moderate pollution, and some monitoring sections in the lower reaches of the East Tiaoxi River are serious pollution, which requires attention (Fig. 4).

**Figure 5:**
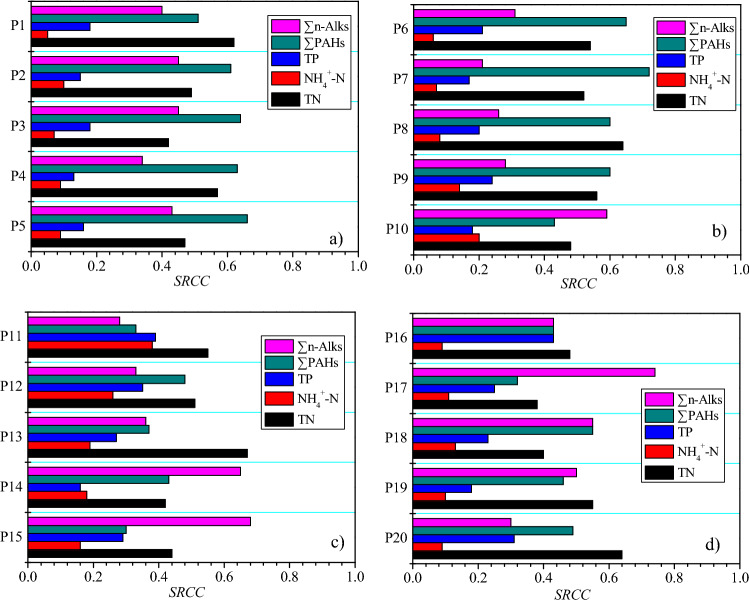
*SRCC* of the 5 evaluation indicators for the 20 monitoring sections in the East Tiaoxi River. (**a**) P1 ~ P5, (**b**) P6 ~ 10, (**c**) P11 ~ P15, (**d**) P16 ~ P20.

The results of *SRCC* analysis are shown in Fig. 5. The higher the *SRCC* value, the greater the influence of the input variable on the target variable. Results show that the water quality parameters that have a greater impact on the comprehensive pollution index in the middle and upper reaches of the East Tiaoxi River are ∑PAHs s and TN. The middle and lower reaches of the East Tiaoxi River are mainly affected by TN and ∑n-Alks. Compared to the upper reaches, the impact of ∑n-Alks on the comprehensive pollution index of lower reaches has increased significantly, which indicates that the impact of shipping on n-alkanes pollution is obvious. Organic pollution in the East Tiaoxi River needs more attention.

**Figure 6:**
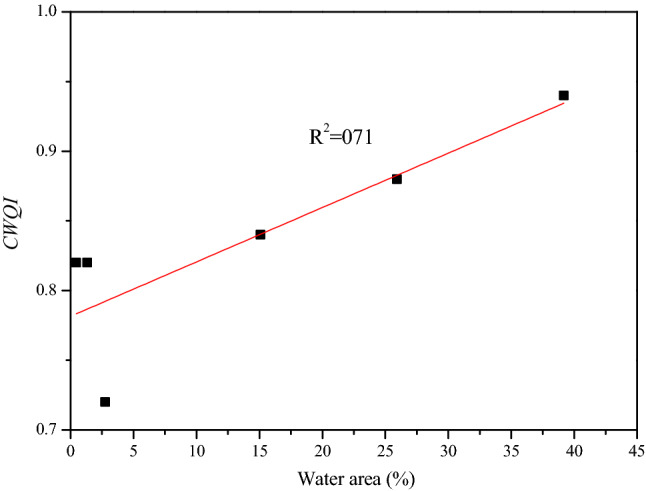
Correlation between the water area and *CWQI* for the catchments with the PAHs as the main pollution factor.

Correlation analysis of the *CWQI* prediction values of 20 sections with the paddy field, dry land, economic forest land, construction land, water area, grassland and other areas of the catchments did not show obvious correlation with a certain land type (correlation coefficients Less than 0.5). Obviously, the pollution sources and pollutant distribution trends of the 20 sections have been affected by multiple factors, and there is no consistent pattern. However, for the cross-sections (P3, P5, P6, P7, P9 and P18) with PAHs as main pollution factor (SRCC > 0.5), the *CWQI* prediction values of these sections have a certain positive correlation with the water area of the catchments, and the correlation coefficient was 0.71(Fig. 6). This shows that the PAHs pollution in East Tiaoxi River may also have a certain relationship with water navigation.

## Conclusions

The concentration levels and spatial–temporal distributions of TN, NH_4_^+^-N, TP, ∑n-Alks and ∑PAHs in the East Tiaoxi River were obtained, which supplemented and improved the database of pollutants in the river and provides some support for East Tiaoxi River water quality management and further improvement plans.

A Monte Carlo-*CWQI* model was established to predict the comprehensive pollution level of the river, two organic pollution indicators, ∑PAHs and ∑n-Alks were incorporate into *CWQI*, and the weight of each pollution indicator in the comprehensive pollution assessment was analyzed by *SRCC* analysis. The established model makes up for the limited data in the sampling study and the insufficient attention to organic pollution in the previous comprehensive pollution assessment methods. The prediction and evaluation results of the model for East Tiaoxi River also provide data and theoretical support for the formulation of PAHs and n-alkanes concentration standards in surface water.

## Supplementary Information

Supplementary Information.

## Data Availability

All data generated or analysed during this study are included in this published article (and its Supplementary Information files).
