# Supplementary material for: Water quality assessment of east Tiaoxi River, China, based on a comprehensive water quality index model and Monte-Carlo simulation
Source: Sci Rep. 2022 Jun 16;12:10042. doi: 10.1038/s41598-022-14293-9 (PMC9203578; doi:10.1038/s41598-022-14293-9)
Supplement: Supplementary file 1 — Supplementary Information. [file 41598_2022_14293_MOESM1_ESM.docx]

**Supplementary Materials**

**Table S1.** Land type and population data for 20 sub-watersheds of East Tiaoxi River

| Sampling  sites | Paddy field（km^2^） | Dry land（km^2^） | Construction land（km^2^） | Woodland（km^2^） | Economic woodland  （km^2^） | Water area（km^2^） | Grassland（km^2^） | Unused land（km^2^） | People（per ten thousand） |
| --- | --- | --- | --- | --- | --- | --- | --- | --- | --- |
| P1 | 71.75 | 2.17 | 30.24 | 459.46 | 16.71 | 10.21 | 19.42 | 0.12 | 6.30 |
| P2 | 12.98 | 0 | 9.83 | 39.30 | 3.95 | 0.27 | 0.45 | 0.17 | 2.60 |
| P3 | 172.64 | 2.46 | 163.26 | 134.19 | 15.70 | 15.09 | 2.12 | 0.08 | 16.70 |
| P4 | 62.05 | 3.15 | 5.73 | 189.73 | 17.17 | 2.59 | 1.19 | 0.19 | 3.30 |
| P5 | 62.53 | 9.92 | 7.75 | 255.60 | 18.43 | 2.75 | 6.47 | 2.18 | 5.63 |
| P6 | 4.51 | 0.31 | 0.57 | 0.22 | 0.60 | 0.45 | 0 | 0 | 0.17 |
| P7 | 6.08 | 1.11 | 2.62 | 8.02 | 2.14 | 1.32 | 0 | 0 | 0.61 |
| P8 | 51.13 | 1.30 | 18.27 | 85.80 | 4.92 | 8.41 | 1.26 | 0 | 2.10 |
| P9 | 145.70 | 2.13 | 57.88 | 160.44 | 7.66 | 25.92 | 1.89 | 0.16 | 0.89 |
| P10 | 3.41 | 0 | 2.92 | 1.98 | 0 | 0.65 | 0 | 0 | 0.24 |
| P11 | 12.78 | 0 | 4.19 | 2.82 | 0.55 | 4.15 | 0.31 | 0 | 0.36 |
| P12 | 253.00 | 0 | 41.66 | 0.96 | 0.09 | 17.13 | 0 | 0 | 0.53 |
| P13 | 22.12 | 0.86 | 2.64 | 7 | 0.41 | 1.69 | 0.30 | 0 | 0.29 |
| P14 | 21.55 | 0.31 | 3.11 | 19.41 | 2.47 | 4.84 | 2.19 | 0.05 | 0.29 |
| P15 | 94.71 | 1.29 | 15.43 | 177.08 | 6.99 | 18.03 | 4.34 | 0.99 | 0.17 |
| P16 | 45.32 | 1.28 | 14.70 | 42.84 | 2.72 | 12.75 | 2.63 | 0 | 0.22 |
| P17 | 56.81 | 0.51 | 16.39 | 1.93 | 0.06 | 16.52 | 1.94 | 0 | 2.05 |
| P18 | 659.11 | 30.25 | 101.47 | 527.48 | 79.89 | 39.19 | 14.54 | 0.48 | 8.85 |
| P19 | 0.89 | 0 | 1.68 | 0 | 0 | 0.33 | 0 | 0 | 3.86 |
| P20 | 31.99 | 1.54 | 13.96 | 8.75 | 0.90 | 9.38 | 0.52 | 0 | 1.25 |

**Table S2.** Probability distributions for the input variables of *CWQI* in East Tiaoxi River

| Sampling sites | Single factor pollution index | Mean | Standard deviation | p | Probability distribution type |
| --- | --- | --- | --- | --- | --- |
| P1 | TN | -0.219 | 0.77 | 0.009 | Normal distribution |
|  | NH_4_^+^-N | -3.421 | 1.08 | 0.038 |  |
|  | TP | -0.975 | 0.47 | 0.084 |  |
|  | ∑PAHs | -0.463 | 0.77 | 0.062 |  |
|  | ∑n-Alks | 0.311 | 0.31 | 0.005 |  |
| P2 | TN | -0.166 | 0.50 | 0.200 | Normal distribution |
|  | NH_4_^+^-N | -3.480 | 1.58 | 0.200 |  |
|  | TP | -0.992 | 0.52 | 0.055 |  |
|  | ∑PAHs | -0.344 | 0.93 | 0.227 |  |
|  | ∑n-Alks | 0.346 | 0.37 | 0.007 |  |
| P3 | TN | -0.116 | 0.54 | 0.200 | Normal distribution |
|  | NH_4_^+^-N | -2.670 | 0.90 | 0.200 |  |
|  | TP | -0.914 | 0.47 | 0.047 |  |
|  | ∑PAHs | -0.541 | 1.13 | 0.126 |  |
|  | ∑n-Alks | 0.327 | 0.38 | 0.013 |  |
| P4 | TN | 0.277 | 0.59 | 0.200 | Normal distribution |
|  | NH_4_^+^-N | -2.314 | 0.96 | 0.200 |  |
|  | TP | -0.993 | 0.51 | 0.028 |  |
|  | ∑PAHs | -0.403 | 1.14 | 0.244 |  |
|  | ∑n-Alks | 0.341 | 0.34 | 0.006 |  |
| P5 | TN | -0.352 | 0.58 | 0.200 | Normal distribution |
|  | NH_4_^+^-N | -3.622 | 0.54 | 0.200 |  |
|  | TP | -1.078 | 0.42 | 0.027 |  |
|  | ∑PAHs | -0.367 | 0.81 | 0.146 |  |
|  | ∑n-Alks | 0.312 | 0.29 | 0.004 |  |
| P6 | TN | -0.124 | 0.61 | 0.200 | Normal distribution |
|  | NH_4_^+^-N | -2.848 | 0.91 | 0.200 |  |
|  | TP | -1.032 | 0.57 | 0.021 |  |
|  | ∑PAHs | -0.266 | 0.83 | 0.293 |  |
|  | ∑n-Alks | 0.306 | 0.23 | 0.001 |  |
| P7 | TN | -0.075 | 0.55 | 0.200 | Normal distribution |
|  | NH_4_^+^-N | -3.058 | 0.91 | 0.200 |  |
|  | TP | -0.948 | 0.43 | 0.030 |  |
|  | ∑PAHs | -0.509 | 1.15 | 0.156 |  |
|  | ∑n-Alks | 0.307 | 0.16 | 0.000 |  |
| P8 | TN | -0.023 | 0.57 | 0.200 | Normal distribution |
|  | NH_4_^+^-N | -2.931 | 0.79 | 0.200 |  |
|  | TP | -0.897 | 0.46 | 0.053 |  |
|  | ∑PAHs | -0.471 | 0.79 | 0.061 |  |
|  | ∑n-Alks | 0.278 | 0.18 | 0.000 |  |
| P9 | TN | -0.135 | 0.70 | 0.200 | Normal distribution |
|  | NH_4_^+^-N | -2.499 | 1.20 | 0.200 |  |
|  | TP | -0.518 | 0.44 | 0.002 |  |
|  | ∑PAHs | -0.432 | 0.94 | -0.143 |  |
|  | ∑n-Alks | 0.353 | 0.24 | 0.000 |  |
| P10 | TN | -0.017 | 0.47 | 0.200 | Normal distribution |
|  | NH_4_^+^-N | -2.350 | 1.28 | 0.200 |  |
|  | TP | -0.585 | 0.29 | 0.033 |  |
|  | ∑PAHs | -0.525 | 0.68 | 0.022 |  |
|  | ∑n-Alks | 0.422 | 0.39 | 0.003 |  |
| P11 | TN | -0.067 | 0.78 | 0.200 | Normal distribution |
|  | NH_4_^+^-N | -0.841 | 1.05 | 0.200 |  |
|  | TP | -0.209 | 0.64 | 0.200 |  |
|  | ∑PAHs | -0.370 | 0.66 | 0.080 |  |
|  | ∑n-Alks | 0.347 | 0.29 | 0.002 |  |
| P12 | TN | 0.177 | 0.52 | 0.200 | Normal distribution |
|  | NH_4_^+^-N | -1.244 | 0.95 | 0.200 |  |
|  | TP | -0.271 | 0.54 | 0.200 |  |
|  | ∑PAHs | -0.631 | 0.94 | 0.042 |  |
|  | ∑n-Alks | 0.336 | 0.30 | 0.003 |  |
| P13 | TN | -0.238 | 1.06 | 0.200 | Normal distribution |
|  | NH_4_^+^-N | -1.156 | 0.69 | 0.200 |  |
|  | TP | -0.368 | 0.52 | 0.200 |  |
|  | ∑PAHs | -0.439 | 0.66 | 0.046 |  |
|  | ∑n-Alks | 0.349 | 0.35 | 0.006 |  |
| P14 | TN | -0.078 | 0.66 | 0.200 | Normal distribution |
|  | NH_4_^+^-N | -1.740 | 1.09 | 0.200 |  |
|  | TP | -0.444 | 0.38 | 0.200 |  |
|  | ∑PAHs | -0.246 | 0.75 | 0.277 |  |
|  | ∑n-Alks | 0.493 | 0.60 | 0.016 |  |
| P15 | TN | 0.177 | 0.52 | 0.200 | Normal distribution |
|  | NH_4_^+^-N | -0.870 | 0.58 | 0.200 |  |
|  | TP | -0.197 | 0.49 | 0.200 |  |
|  | ∑PAHs | -0.361 | 0.62 | 0.068 |  |
|  | ∑n-Alks | 0.523 | 0.61 | 0.013 |  |
| P16 | TN | -0.179 | 0.84 | 0.200 | Normal distribution |
|  | NH_4_^+^-N | -0.957 | 0.38 | 0.200 |  |
|  | TP | 0.042 | 0.64 | 0.200 |  |
|  | ∑PAHs | -0.480 | 0.96 | 0.111 |  |
|  | ∑n-Alks | 0.487 | 0.45 | 0.003 |  |
| P17 | TN | -0.040 | 0.50 | 0.200 | Normal distribution |
|  | NH_4_^+^-N | -1.503 | 0.54 | 0.200 |  |
|  | TP | -0.554 | 0.53 | 0.200 |  |
|  | ∑PAHs | -0.539 | 0.66 | 0.017 |  |
|  | ∑n-Alks | 0.532 | 0.57 | 0.007 |  |
| P18 | TN | -0.109 | 0.51 | 0.200 | Normal distribution |
|  | NH_4_^+^-N | -1.469 | 0.58 | 0.200 |  |
|  | TP | -0.504 | 0.44 | 0.200 |  |
|  | ∑PAHs | -0.408 | 0.87 | 0.132 |  |
|  | ∑n-Alks | 0.473 | 0.41 | 0.002 |  |
| P19 | TN | -0.212 | 0.75 | 0.200 | Normal distribution |
|  | NH_4_^+^-N | -1.361 | 0.46 | 0.200 |  |
|  | TP | -0.510 | 0.37 | 0.200 |  |
|  | ∑PAHs | -0.660 | 0.91 | 0.029 |  |
|  | ∑n-Alks | 0.469 | 0.38 | 0.002 |  |
| P20 | TN | -0.165 | 0.69 | 0.200 | Normal distribution |
|  | NH_4_^+^-N | -1.339 | 0.36 | 0.200 |  |
|  | TP | -0.626 | 0.53 | 0.200 |  |
|  | ∑PAHs | -0.534 | 0.72 | 0.026 |  |
|  | ∑n-Alks | 0.373 | 0.19 | 0.000 |  |

**Fig. S1.** Annual average concentration distribution of the 33 n-alkanes in monitoring sections P2, P15 and P20 (2018.10-2019.9)
